# Detection of selected pathogens in reproductive tissues of wild boars in the Campania region, southern Italy

**DOI:** 10.1186/s13028-024-00731-3

**Published:** 2024-03-05

**Authors:** Gianmarco Ferrara, Nadia Piscopo, Ugo Pagnini, Luigi Esposito, Serena Montagnaro

**Affiliations:** Department of Veterinary Medicine and Animal Productions, University of Naples ‘‘Federico II’’, Via Delpino no. 1, 80137 Naples, Italy

**Keywords:** *Brucella*, *Coxiella*, Molecular detection, Porcine circovirus, Porcine parvovirus, Pseudorabies, Reservoir, Wild boar

## Abstract

Monitoring disease among wildlife is critical to preserving health in both domestic animals and wildlife, and it becomes much more critical when the diseases cause significant economic damage to the livestock industry or threaten public health. Given the continuous increase in populations and its role as a reservoir for several infections, wild boar (*Sus scrofa*) requires special attention regarding disease surveillance and monitoring. In this study, we investigated the molecular prevalence of selected pathogens in the wild boar population of Campania, southern Italy. The prevalence of pathogens causing reproductive problems in pigs (*Sus domesticus*), including porcine parvovirus (PPV), porcine circovirus types 2 and 3 (PCV-2 and PCV-3), pseudorabies virus (PRV), *Coxiella burnetii*, and *Brucella suis*, was evaluated by testing the reproductive organs collected from 63 wild boars with polymerase chain reaction. The most common pathogens were PPV (44.4%) and two porcine circoviruses (14.3%). PRV and *C. burnetii*, on the other hand, showed a significantly lower prevalence (1.6%). No reproductive organs tested were positive for *B. suis*. Risk factor analysis revealed a correlation between age and PCV-2 positivity, with animals less than 12 months old having significantly higher prevalence rates.

Our findings suggest that wild boars hunted in the Campania region harbour several infections potentially transmissible to other mammals’ reproductive tracts. Furthermore, our results emphasized the importance of strict adherence to biosecurity protocols on domestic swine farms, especially on free-range farms, to avoid interactions between domestic and wild animals.

**Supplementary Information:**

The online version contains supplementary material available at 10.1186/s13028-024-00731-3.

## Findings

Reproductive failure in pigs (*Sus domesticus*) has significant implications for the swine industry, especially when it occurs endemically, as, for example, when it is caused by infectious diseases. In addition to economic concerns, reproductive failure in pigs may have a relevant impact on human health when caused by zoonotic pathogens [1, 2]. While it is possible to monitor and prevent these infections in domestic animals through eradication plans, immunizations, and biosecurity measures, achieving so in wildlife is considerably more difficult. Wildlife species can serve as reservoirs for pathogens responsible for reproductive failure in other wildlife species, domestic animals, and humans, and at the same time, they can act as a sentinel for the presence of a specific infection in a specific geographical area [1]. Wild boar (*Sus scrofa*) is one of the most abundant wildlife species for which monitoring is critical due to its population growth in Europe and its role in the epidemiology of infectious diseases considered impactful in domestic swine [3]. As a result, contact between wild boars and domestic pigs is becoming more common, especially when they share the same environment (as in free-range pig farming) [2].

There are several pathogens described as responsible for reduced fertility, which can be caused by a variety of conditions, including stress, environmental variables [1, 4, 5]. Porcine parvovirus (PPV) is a primary pathogen responsible for SMEDI (Stillbirth, Mummification, Embryonic Death, and Infertility), and its spread has been described in both domestic and wild pigs [6]. Porcine circovirus 2 (PCV-2), the causative agent of postweaning multisystemic wasting syndrome (PMWS), and porcine circovirus 3 (PCV-3) are other DNA viruses described worldwide as responsible for respiratory and enteric diseases as well as reproductive disorders [7]. Pseudorabies virus (PRV), also called Suid Herpesvirus type 1 (SHV-1), in addition to inducing mortality in neonatal piglets, is also one of many pathogens responsible for reproductive failure in sows [8]. Among the bacterial pathogens capable of colonizing the reproductive system of wild boar are *Brucella suis* and *Coxiella burnetii*, two bacteria with a zoonotic potential as well as causing extensive damage to farms [9, 10].

All these pathogens have been detected in European wild boar populations and can be transmitted to other swine via different routes of transmission such as the respiratory route for PCV and PRV or the faecal-oral cycle for PPV, and through the reproductive route. In contrast to domestic animals, which are routinely tested for the aforementioned pathogens, information on the presence of thesepathogens in wildlife is limited.

The aim of this work was to evaluate the presence of pathogens of veterinary and public health interest in the reproductive organs of apparently healthy wild boars hunted in the Campania region, southern Italy.

Reproductive organs (uterus and ovaries in females and testicles in males) were collected from wild boars harvested during two hunting seasons 2021–2023 (no animal was specifically sacrificed for this study). A total of 63 samples (originating from 37 males and 26 females, whose ages in months were estimated using tooth eruption patterns) were stored at -80 °C prior to processing [11]. DNA extraction from 25 mg of tissue was performed using a commercial column-based kit and a TissueLyser, following the manufacturer’s instructions (DNeasy Blood & Tissue Kits, QIAGEN; TissueLyser LT, QIAGEN, Germany). After quantification with Nanodrop, 50 ng of DNA were used as templates for specific polymerase chain reactions previously described in the literature. Suppl. Table 1 summarizes the primers, target regions, sizes, and references used in this study [10, 12–15]. To confirm DNA integrity, each sample was tested for detection of the porcine β-actin gene [12]. Generally, real-time PCR was performed in a volume of 20 µl using SYBR Green (Biorad, US), whereas endpoint PCR was performed in a volume of 50 µl using HotStarTaq DNA Polymerase (QIAGEN, Germany) as previously described [12–15]. The detection of *C. burnetii* was performed using iTaq Universal Probes Supermix (Biorad, US) [15]. PCR products were analyzed in real-time or visualized in a 2% electrophoresis agarose gel. Positive controls were represented by samples positive in previous research for PCV-2, PCV-3, and *B. suis*. DNA extracted from Suid herpesvirus 1 - VR-135 (ATCC), Porcine parvovirus NADL-2 - VR-742 (ATCC) and *C. burnetii* Nile Mile RSA 493 PhI strain were additional controls [16, 17]. Statistical analysis was performed using Chi square test and Fisher’s exact test with JMP Pro version 15.0.0 (SAS Institute Inc.).

A total of 14.3% of the animals tested positive for PCV-2, the same percentage for PCV-3, and 44.4% for PPV, suggesting that it was the most frequent virus in the reproductive tissues of wild boars hunted in Campania. PRV and *Coxiella* DNA were detected only in 1.6% of samples (Table 1) while no reproductive organs tested were positive for *B. suis*. Only two animals tested positive for both PPV and PCV-2, six for PPV and PCV-3, one for both circoviruses, and one for PPV and *Coxiella*, indicating a moderate occurrence of co-infection (17.5%). PPV, PCV-2, and PCV-3 were all detected in one animal. Because only 30 of the 63 animals tested negative for all the pathogens examined, more than half of the animals harboured at least one pathogen in their reproductive systems. Statistical analysis (chi square test) highlighted a greater frequency (34.9%) of single infections compared to co-infections (χ^2^ = 4.97; *p* = 0.025).

**Table 1:** Prevalence of selected pathogens in reproductive organs of wild boars

Factor		Positive	%	95%CI	χ^2^	p
PCV-2		9/63	14.3	5.6–22.9		
PCV-3		9/63	14.3	5.6–22.9		
PPV		28/63	44.4	32.2–56.7	41.3	< 0.001
PRV		1/63	1.6	0–4.7		
*Coxiella burnetii*		1/63	1.6	0–4.7		
*Brucella suis*		0/63	0			

legend: Prevalence of porcine circovirus-2 (PCV-2), porcine circovirus-3 (PCV-3), porcine parvovirus (PPV), pseudorabies virus (PRV), *Coxiella burnetii* and *Brucella suis*. For each pathogen, the number of positive animals out of the total, the % prevalence, and the 95% confidence intervals are reported. Chi-square (**χ**^**2**^) test revealed that the most frequently identified pathogen was PPV (*p* value lower than 0.001)

During the risk factor evaluation, we found a significant variation depending on age for PCV-2, with younger animals being more likely to test positive (Table 2). Even if it was not statistically significant, PCV-3 appeared to be more common in males.

**Table 2:** Risk factor evaluations for PCV-2, PCV-3 and PPV positivity in wild boars

		PCV-2				PCV-3				PPV			
***Factor***	*n*	*positive*	*%*	*95% CI*	*p*	*positive*	*%*	*95% CI*	*p*	*positive*	*%*	*95% CI*	*p*
**Total**	63	9	14.3	5.6–22.9		9	14.3	5.6–22.9		28	44.4	32.2–56.7	
**Years**													
2021	50	6	12.0	2.9–21.0		6	12.0	2.9–21.0		23	46.0	32.2–59.8	
					0.375				0.375				0.757
2022	13	3	23.1	0.17–45.9		3	23.1	0.17–45.9		5	38.4	12.0–64.9	
**Age (months)**													
0–12	16	5	31.2	8.5–53.9		4	25	3.7–46.2		7	43.7	19.4–68.1	
					**0.022**				0.21				1.00
> 12	47	4	8.5	0.53–16.4		5	10.6	1.8–11.5		21	44.7	30.5–58.9	
**Sex**													
M	37	5	13.5	2.5–24.5		8	21.6	8.4–34.9		20	54.1	38.0–70.1	
					1.00				0.066				0.077
F	26	4	15.4	1.52–29.2		1	3.8	0.00–11.2		8	30.7	13.0–48.5	
**Location**													
Avellino	50	8	16	5.8–26.2		8	16	5.8–26.2		25	50.0	36.1–63.9	
					0.67				0.093				0.118
Benevento	13	1	7.7	0.00–22.2		1	7.7	0.00–22.2		3	23.1	0.2–45.9	

legend: Risk factor evaluations for porcine circovirus-2 (PCV-2), porcine circovirus-3 (PCV-3) and porcine parvovirus (PPV). For each pathogen, the number of positive animals out of the total, the % prevalence, and the 95% confidence intervals are reported. Fisher’s exact test revealed an higher PCV-2 prevalence for young wild boars

The results obtained in our study conform to those described in the literature for common viruses found in wild boars (PCV and PPV). In fact, serological and molecular evidence of these viruses has been routinely described worldwide, reporting seroprevalences up to 100% and molecular positivity oscillating between 40% and 70% (depending on the type of matrix tested) [7, 18]. Studies identifying circovirus DNA in tonsils and spleens usually tend to have higher prevalence rates [16]. In our investigation, we observed a correlation between young age and PCV-2 positivity. This finding is controversial, as previous studies have found greater prevalence in adults, particularly in multiparous domestic sows. However, considering the pressures to which wild animals are subjected in the wild, debilitating and predisposing factors to infections may impact younger individuals more. The differences observed between sexes could be related to the different colonization patterns of this virus in the male and female genital tracts rather than epidemiological variables [16, 18].

High prevalences have also been described for PPV in the European wild boar population (ranging from 24 to 78% during serological surveys and around 20% using molecular approaches) [19]. The frequent occurrence of these infections in wild boar is also described in other countries that are major pork producers, such as China, Korea, and India, also in this case with high prevalence, and in some cases, highly virulent strains have been isolated [20]. The pathogenic role of these viruses is still debated since, even if isolated in significant percentages in aborted foetuses, they have usually been detected in apparently healthy animals as well. A study carried out in Argentina found that 17 out of 131 aborted foetuses were PPV-positive [6]. A large-scale study identified PCV-2 in 42.7% of cases, PPV in 6.2%, and PRV in only 1.8% of stillbirths using the same approach [21].

The pathogenetic role of PRV during pregnancy in wild boars and the existence of an additional route of transmission have already been demonstrated in a previous study that described high seroprevalence for PRV and identified its gB sequence in aborted fetuses in wild boars [22]. The low prevalence did not represent the real Italian epidemiological situation for PRV (widespread in the whole country in wildlife) and should depend on the target organ we used; in fact, usually higher prevalences are identified when using tissues where the virus becomes latent (such as the trigeminal and sacral ganglia) or vaginal swabs [8, 23]. Evidence in the literature describes PCV-2 and PCV-3 co-infections as common, as well as the co-infections between PPV and circoviruses [16, 24].

The detection of *C. burnetti* in wild boar testes was surprising. These wild animals, like others, can become infected both per os or by the respiratory route (due to the microorganisms eliminated in the external environment by infected animals) and through competent species of ticks [25]. Although the pathogenesis and symptomatology of *Coxiella* in swine are still debated, these animals are certainly susceptible to the infection and could, based on comparative aspects, transmit the infection transgenitally [9, 26].

On the other hand, the absence of animal testing positive for *Brucella suis* should not be considered a surprising result, although infection has been demonstrated in recent studies in wild boars in southern Italy, colonization of the reproductive tract by *Brucella* is limited to bacteraemia in the male and pregnancy in the female [10, 27].

In recent years, the presence of wild boar has been considered a risk to the health of domestic animals and humans. The concomitant increase in free-ranging pigs has increased the risk of disease transmission between wild boars and domestic pigs. Many pathogens that cause infectious diseases in domestic pigs may also be found in wild boars as the wild progenitor of domestic pigs. To prevent the transmission of disease from wild boars to domestic pigs, strict prevention and control measures, such as continuous wildlife disease surveillance and strategic depopulation methods, should be implemented.

### Electronic supplementary material

Below is the link to the electronic supplementary material.

Supplementary Material 1

## Data Availability

The datasets used and/or analysed during the current study are available from the corresponding author on reasonable request.

## Declarations

### Prior publication

Data have not been published previously.
