# Supplementary material for: Detection of selected pathogens in reproductive tissues of wild boars in the Campania region, southern Italy
Source: Acta Vet Scand. 2024 Mar 5;66:9. doi: 10.1186/s13028-024-00731-3 (PMC10916309; doi:10.1186/s13028-024-00731-3)
Supplement: Supplementary file 1 — Supplementary Material 1 [file 13028_2024_731_MOESM1_ESM.docx]

| **Gene** |  | **Forward (5’-3’)** | **Reverse (5’-3’)** | **Size (bp)** | **Ref** |
| --- | --- | --- | --- | --- | --- |
| β-Actin |  | CACGCCATCCTGCGTCTGGA | AGCACCGTGTTGGCGTAGAG | 100 | Nygard et al. |
| VP2 (PPV) |  | GGGCTTGGTTAGAATCAC | TGGTGGTGAGGTTGCTGAT | 313 | Zheng et al. |
| ORF-2 (PCV-2) |  | ATGGCG GGAGGAGTAGTTT | CCCTTTGAATACTACAGCG | 171 | Zheng et al. |
| Rep (PCV-3) |  | GCTACGAGTGTCCTGAAGATAAG | GCCTCCACACTCCACAATAG | 138 | Tian et al. |
| gE (PRV) |  | TCTCGGACATGGGCGACT | ACGTAGTACAGCAGGCAC | 92 | Tian et al. |
| IS1111 (*Coxiella burnetii*)^#^ |  | GTCTTAAGGTGGGCTGCGTG | CCCCGAATCTCATTGATCAGC | 295 | Klee et al. |
| IS711 |  | GGCATGAACCGCTGTCC | CTTCCGGGGCGAGTTG | 141 | Pilo et al. |

^#^In this real time-PCR also a Taq man probe was used FAM-AGCGAACCATTGGTATCGGACGTT-TAMRA-TATGG
